# Adherent Monomer-Misfolded SOD1

**DOI:** 10.1371/journal.pone.0003497

**Published:** 2008-10-23

**Authors:** Yasuhiro Watanabe, Eri Morita, Yasuyo Fukada, Koji Doi, Kenichi Yasui, Michio Kitayama, Toshiya Nakano, Kenji Nakashima

**Affiliations:** Department of Neurology, Institute of Neurological Sciences, Faculty of Medicine, Tottori University, Yonago, Japan; University of Giessen, Germany

## Abstract

**Background:**

Multiple cellular functions are compromised in amyotrophic lateral sclerosis (ALS). In familial ALS (FALS) with Cu/Zn superoxide dismutase (SOD1) mutations, the mechanisms by which the mutation in SOD1 leads to such a wide range of abnormalities remains elusive.

**Methodology/Principal Findings:**

To investigate underlying cellular conditions caused by the SOD1 mutation, we explored mutant SOD1-interacting proteins in the spinal cord of symptomatic transgenic mice expressing a mutant SOD1, SOD1^Leu126delTT^ with a FLAG sequence (DF mice). This gene product is structurally unable to form a functional homodimer. Tissues were obtained from both DF mice and disease-free mice expressing wild-type with FLAG SOD1 (WF mice). Both FLAG-tagged SOD1 and cross-linking proteins were enriched and subjected to a shotgun proteomic analysis. We identified 34 proteins (or protein subunits) in DF preparations, while in WF preparations, interactions were detected with only 4 proteins.

**Conclusions/Significance:**

These results indicate that disease-causing mutant SOD1 likely leads to inadequate protein-protein interactions. This could be an early and crucial process in the pathogenesis of FALS.

## Introduction

Amyotrophic lateral sclerosis (ALS), a progressive and fatal disorder of the central nervous system (CNS), selectively affects both upper and lower motor neurons in the cerebral cortex, brain stem and spinal cord. Approximately 10 percent of ALS cases are of the hereditary type (familial ALS; FALS), and about 20% of FALS cases are associated with Cu/Zn superoxide dismutase (SOD1) mutations [1], [2]. Since the first report of a link between the SOD1 mutations and FALS [3], more than 130 different mutations have been reported [1], [2]. Two leading hypotheses have been advanced to explain the apparent “toxic gain of function” of the mutant SOD1 protein [1], [2]. The first of these, the “aggregation toxicity” hypothesis, suggests that mutant SOD1 becomes misfolded and oligomerized to form intracellular aggregates, which then diminish the availability of essential proteins for normal cellular function. The second hypothesis, the “oxidative damage” theory, conjectures that toxicity is caused by the aberrant chemistry of the metal-binding sites of the mutant SOD1, such as peroxidase or superoxide-reducing activities and peroxynitrite catalysis. These hypotheses, however, are unable to explain the multiple perturbations of cellular function identified in FALS, including excessive excitatory toxicity, protein misfolding, impaired energy production, abnormal calcium metabolism, altered axonal transport, activation of proteases and nucleases, and so on [1], [2]. Furthermore non-neural cell populations substantially contribute to motor neuron degeneration [4]. So far there is no single pathway that explains such diverse cellular consequences.

To investigate the nature of the mutant SOD1, we have studied a SOD1 mutation characterized by a 2-bp deletion at codon 126 (SOD1^Leu126delTT^) [5], [6]. The mutation causes a frame shift and results in a premature stop codon. The SOD1^L126delTT^ consists of only 130 amino acids, compared to the 153 found in the wild type [7]. The SOD1^L126delTT^ does not form a functional dimer as it does in the case of normal SOD1 [8]. As a consequence, the quantity of the SOD1^L126delTT^ mutant is a mere trace in patients with this mutation [6], [9]. This observation also holds true for overexpression of SOD1^L126delTT^ in transgenic mice [10]. Our particular questions are how more than 130 different SOD1 mutations all result in motor neuron degeneration, and how a low level of mutant SOD1 expression, like SOD1^L126delTT^ leads to FALS in a similar manner to high level expression that is seen with point mutations. Importantly, homodimeric mutant SOD1 forms unnatural, partially folded monomeric and soluble oligomeric intermediates before aggregation *in vitro* [11] and *in vivo* [12]. Moreover, the soluble fibrous oligomers could be far more toxic than the visible inclusions [13]. If a small population of monomer-misfolded SOD1 is pathogenic in FALS, analysis should be difficult when using homodimeric mutant SOD1 like the mutant SOD1^G93A^ as the majority of homodimeric protein has merely a bystander role. Meanwhile transgenic mice expressing the SOD1^L126delTT^ could be an ideal model for analyzing the disease process of FALS as the SOD1^L126delTT^ protein is inherently monomeric. This thus enables the important capability of seeking the proteins that specifically interact with mutant SOD1. Using a pull-down proteomic method, we present here that many inadequate protein-protein interactions are seen in the SOD1^L126delTT^.

## Results

### Proteomic Analyses

**Figure 1:**
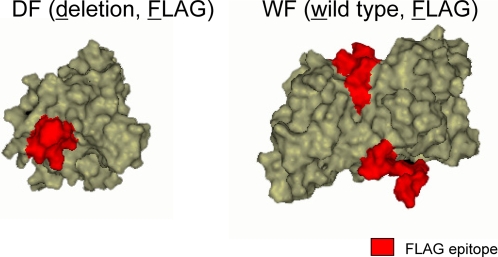
Computer assisted simulation of the FLAG-tagged SOD1 proteins. Simulation models of the FLAG-tagged SOD1s were constituted using Discovery Studio 1.7 software (Accelrys, USA) from Protein Data Bank (PDB) data; 1MFM for DF, 2V0A for WF as templates. The red parts indicate the FLAG epitopes. The DF mutant SOD1 possibly exposes a hydrophobic structure, which is normally buried by the dimer interface.

Two lines of transgenic mice were used in this experiment. The DF (deletion, FLAG) mice ubiquitously expressed the SOD1^L126delTT^ with a FLAG sequence at *C* terminal, as did the WF (wild type, FLAG) mice exhibiting a wild-type human SOD1 with the FLAG sequence [10] (Fig. 1). The DF mice showed ALS-like symptoms, while the WF mice did not. As a further negative control, wild-type C57BL/6 (NTG) mice were subjected to the same analysis. The tissues were solublized, and the FLAG-tagged SOD1 and cross-linking proteins were selectively enriched using a FLAG affinity purification system. After digestion in a single tube, the samples were subjected to liquid chromatography-tandem mass spectrometry (LC-MS/MS) analysis, whereupon each protein was identified after reference to a protein database. Proteins whose peptide hit score was less than 2 were eliminated. In symptomatic DF mice aged 145 days, 34 proteins (or subunits) were unambiguously identified by the analysis (Table 1). These proteins could be grouped into 7 approximate functional categories: heat shock proteins (HSPs) and protein degradation; ATPases; glycogenolysis, glycolysis and TCA cycle; cytoskeleton and structure; membrane and protein trafficking; protein biosynthesis; and others (Table 1). Solute carrier family 25 (SLC25) proteins (members 4 and 5) categorized as “others” were involved in ATP transport from mitochondria to cytosol [14]. In the WF analysis identified proteins were mouse SOD1, keratin and 2 of the heat shock protein 70 member proteins. Consistent with our previous observation, the WF SOD1 interacted with an intrinsic mouse SOD1 during dimer formation, whilst the DF did not. A mere 3 proteins were singled out in the NTG mice and of these only β tubulin overlapped with the DF mouse set.

**Table 1:** Identification of SOD1-interacting proteins in spinal cord of DF, WF and NTG mice aged 145 days.

DF					WF				NTG			
Protein	Abbreviation	Accession no.	Probability score	No. of peptide	Protein	Accession no.	Probability score	No. of peptide	Protein	Accession no.	Probability score	No. of peptide
**HSPs and protein degradation**												
heat shock cognate 70	Hsc70, HspA8	13242237	170.22	20	heat shock cognate 70	13242237	30.17	3				
heat shock protein 70-2	Hsp70-2, HspA2	123621	80.25	10	heat shock protein 70-2	123621	30.16	3				
heat shock 70 protein 1-like	Hapa1L	56757584	40.25	4								
heat shock protein 9A	HspA9A	6754256	30.16	3								
ubiquitin carboxy-terminal hydrolase L1	UCHL1	61098212	20.24	3								
**ATPases**												
Na/K ATPase α 2 subunit	ATP1A2	30409956	120.19	12								
Na/K ATPase α 3 subunit	ATP1A3	27552786	100.20	10								
Na/K ATPase α 1 subunit	ATP1A1	21450277	40.18	4								
ATP synthase β subunit	ATP5B	31980648	80.25	9								
ATP synthase α subunit	ATP5A	6680748	50.20	5								
ATP synthase γ subunit	ATP5C	11602916	30.17	3								
**Glycogenolysis, glycolysis, and TCA cycle**												
α (non-neuron) enolase*	ENO1	12963491	70.25	7					pyruvate kinase	2506796	50.22	5
lactate dehydrogenase	LDH	6678674	60.22	6								
brain glycogen phosphorylase*	PYGB	24418919	30.25	3								
phosphofructokinase 1	PFKA, PFKM	13638207	30.20	3								
malate dehydrogenase 1	MDH1	31982178	30.17	3								
aconitase 2	ACO2	18079339	30.16	3								
glyceraldehyde-3-phosphate dehydrogenase	GAPDH	6679937	20.33	3								
**Cytoskeleton and structure**												
α tubulin	TUBA	34740335	140.22	14	keratin	6678643	30.21	3	keratin	627899	50.21	6
β tubulin	TUBB	5174735	120.25	13					β actin	4501885	30.21	3
β actin	ACTB	49868	60.20	6								
cofilin 1	CFL1	6680924	50.26	5								
microtubule-associated protein 2	MAP2	126741	50.23	5								
myelin basic protein*	MBP	6754658	30.16	3								
**Membrane and protein trafficking**												
syntaxin binding protein 1	STXBP1	21594764	40.19	5								
clathrin heavy chain	CLTC	51491845	40.20	4								
**Protein biosynthesis**												
eukaryotic translation elongation factor 1	eEF1A	51873060	40.17	4								
**Others**												
2′,3′-cyclic-nucleotide 3′-phosphodiesterase I*	CNP	2160434	80.19	8	mouse Cu/Zn superoxide dismutase	45597447	20.22	3				
dihydropyrimidinase-like 2	DPYSL2	40254595	50.20	5								
latexin	LXN	31980632	40.25	5								
solute carrier family 25, member 4	SLC25A4	82917335	40.22	4								
solute carrier family 25, member 5	SLC25A5	22094075	30.22	3								
solute carrier family 25, member 12	SLC25A12	27369581	30.21	4								
glutamate oxaloacetate transaminase 2	GOT2	6754036	40.21	4								

* Proteins expressed in non-neural cells.

To confirm if region and age specificities existed in the DF mice, a proteomic profile of spinal cords aged 145 days was compared with similar profiles of spinal cords aged 35 days (presymptomatic) and the cerebellum aged 145 days (Table 2). The results indicated that the interacting protein groups were essentially similar to each other, while the number of interacting proteins in the spinal cord age 145 days exceeded that of the others. Ubiquitin carboxy-terminal hydrolase L1 (UCHL1), ATP synthase β and γ subunits, α (non-neuron) enolase (Eno1), cofilin 1, latexin and SLC25 members, along with some other proteins were specific for the symptomatic DF spinal cords (Table 2). Intriguingly, heat shock protein 70 (Hsp70), a stress-inducible form of HSPs, was absent only in the symptomatic DF spinal cords. Meanwhile, possessing over 85% homology with Hsp70, constitutively expressed housekeeping chaperone heat shock cognate 70 (Hsc70) was recognizable in all samples (confusing terminology and the function of Hsp70 family proteins are reviewed by Daugaard et al [15]).

**Table 2:** Summary of proteins identified in spinal cord and cerebellum of DF mice.

DF (35) spinal cord				DF (145) spinal cord*	DF (145) cerebellum			
Protein	Accession no.	Probability score	No. of peptide	Protein	Protein	Accession no.	Probability score	No. of peptide
**HSPs and protein degradation**								
heat shock cognate 70	13242237	200.28	22	heat shock cognate 70	heat shock cognate 70	13242237	160.25	17
heat shock protein 70-2	123621	80.27	9	heat shock protein 70-2	heat shock protein 70-2	123621	70.26	7
heat shock protein 70***	56757667	40.25	4	heat shock 70 protein 1-like	heat shock protein 70***	56757667	40.24	4
				heat shock protein 9A**	heat shock 70 protein 1-like	56757584	30.26	3
				ubiquitin carboxy-terminal hydrolase L1**				
**ATPases**								
Na/K ATPase α 3 subunit	27552786	90.26	10	Na/K ATPase α 2 subunit	Na/K ATPase α 3 subunit	27552786	100.23	11
Na/K ATPase α 2 subunit	30409956	80.26	9	Na/K ATPase α 3 subunit	Na/K ATPase α 2 subunit	30409956	90.23	10
Na/K ATPase α 1 subunit	21450277	60.26	7	Na/K ATPase α 1 subunit	Na/K ATPase α 1 subunit	21450277	80.23	9
Na/K ATPase α 4 subunit***	18203577	20.21	3	ATP synthase β subunit**	Na/K ATPase α 4 subunit***	18203577	20.22	3
ATP synthase α subunit	6680748	40.30	4	ATP synthase α subunit	ATP synthase α subunit	6680748	70.25	7
				ATP synthase γ subunit**	potassium-transporting ATPase α subunit	20137339	30.19	3
**Glycogenolysis, glycolysis, and TCA cycle**								
glyceraldehyde-3-phosphate dehydrogenase	6679937	60.34	7	α enolase (non-neuron)**	glyceraldehyde-3-phosphate dehydrogenase	6679937	40.36	5
				lactate dehydrogenase**	pyruvate kinase	551295	40.23	4
				brain glycogen phosphorylase**	aconitase 2	18079339	40.27	4
				phosphofructokinase 1**				
				malate dehydrogenase 1**				
				aconitase 2				
				glyceraldehyde-3-phosphate dehydrogenase				
**Cytoskeleton and structure**								
β tubulin	12963615	80.34	9	α tubulin	β tubulin	5174735	90.37	11
α tubulin	34740335	70.28	7	β tubulin	α tubulin	34740335	70.29	8
keratin***	112983636	30.20	4	β actin	β actin	49868	30.22	3
β actin	4501885	40.20	4	cofilin 1**	keratin***	54607171	20.21	3
myelin basic protein	6754658	30.19	3	microtubule-associated protein 2**				
				myelin basic protein				
**Membrane and protein trafficking**								
clathrin heavy chain	51491845	30.25	3	syntaxin binding protein 1	syntaxin binding protein 1	21594764	40.21	4
				clathrin heavy chain	clathrin heavy chain	51491845	30.26	3
**Protein biosynthesis**								
eukaryotic translation elongation factor 1	51873060	30.19	4	eukaryotic translation elongation factor 1	eukaryotic translation initiation factor 4B***	55976513	50.24	6
eukaryotic translation initiation factor 4B***	55976513	30.22	3					
**Others**								
2′,3′-cyclic-nucleotide 3′-phosphodiesterase I	2160434	90.27	10	2′,3′-cyclic-nucleotide 3′-phosphodiesterase I	dihydropyrimidinase-like 2	40254595	40.30	4
dihydropyrimidinase-like 2	40254595	40.30	4	dihydropyrimidinase-like 2	adenylate cyclase-associated protein 2	13385554	40.28	4
				latexin**	protein phosphatase 1B	33859600	40.30	4
				solute carrier family 25, member 4**				
				solute carrier family 25, member 5**				
				solute carrier family 25, member 12**				
				glutamate oxaloacetate transaminase 2**				

* Data are same as Table 1. ** Proteins specific for DF (145). *** Proteins absent only in DF (145).

### Immunoprecipitation and western analyses

**Figure 2:**
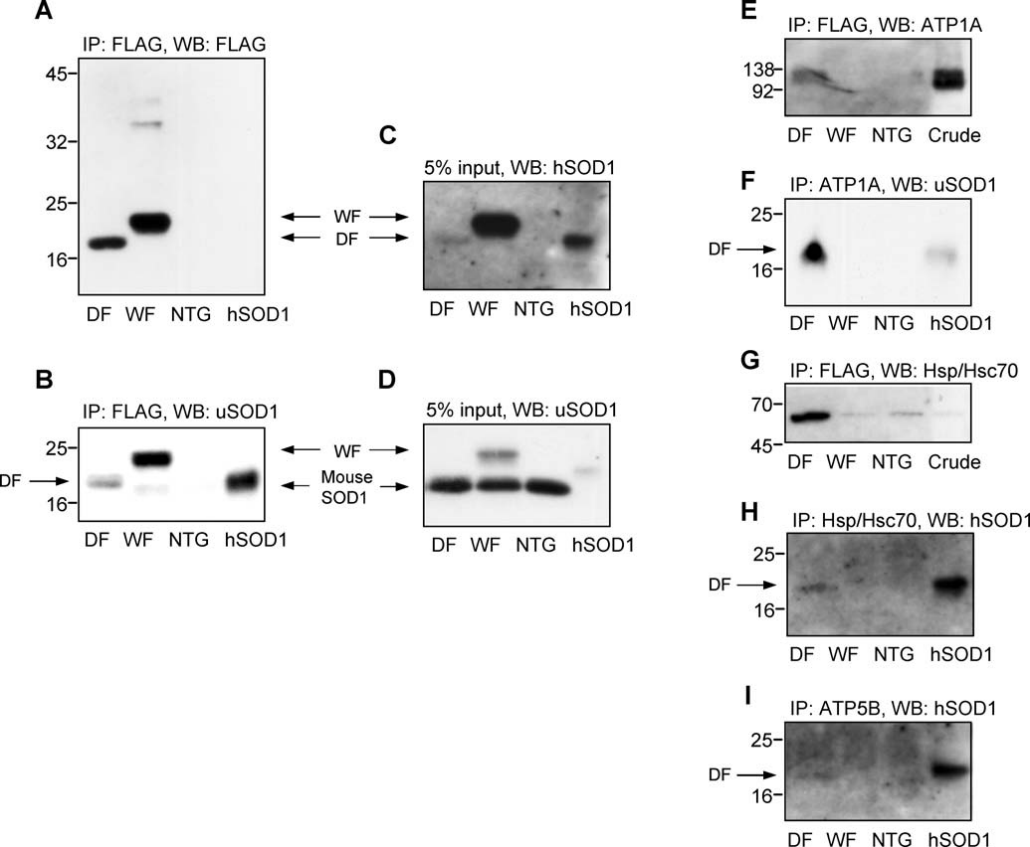
Immunoprecipitation and western blot analyses of mouse spinal cords. The FLAG-tagged SOD1 and the ligand proteins were immunoprecipitated using the FLAG affinity resin and detected by the anti FLAG antibody (A) and anti-SOD1 polyclonal antibody (uSOD1) that recognizes both human and mouse SOD1 (B). The DF mutant SOD1 shows a distinct single band (A and B). In contrast, the WF shows high molecular weight bands (A) and a low molecular weight band which is possibly the mouse intrinsic SOD1 (B), indicating that the WF forms the heterodimer with the mouse SOD1. Crude spinal cord extracts (5% input) were analyzed using antibodies for human specific SOD1 (hSOD1) (C) and for uSOD1 (D). Compared to the WF, the amount of the DF SOD1 protein is considerably lower (C). The WF itself is lower still compared to the mouse intrinsic SOD1 (D). The immunoprecipitation products with the FLAG were analyzed by antibodies for ATP1A (E) and Hsp/Hsc70 (G). In this analysis, only the DF preparations show the presence of ATP1A (E). In the Hsp/Hsc70 analysis in (G), there are non-specific precipitations also in WF and NTG. In contrast, the immunoprecipitated samples for ATP1A (F), Hsp/Hsc70 (H) and ATP5B (I) were analyzed using antibodies for hSOD1 (F) or uSOD1 (H and I). Correspondingly, the human form of SOD1 is only seen to be co-immunoprecipitated in DF preparation (F, H and I). Lanes from the left are: DF, WF, NTG, and a purified human SOD1 (A, B, C, D, F, H, and I); and DF, WF, NTG, and a crude soluble fraction of NTG mice (E and G).

The DF mutant SOD1 represents a relatively small proportion of the total SOD1 content of the spinal cord compared to both the WF and wild-type mouse SOD1s (Fig. 2A–C). WF SOD1 interacts with mouse intrinsic SOD1 (Fig. 2B). Among the DF-mutant SOD1-interaction proteins (subunits), we especially focused on interactions with the Na/K ATPase subunit α (ATP1A) and ATP synthase β subunit (ATP5B). This is not only because they showed high hit scores in the proteomic analysis, but also because they are among the most important proteins necessary for cellular energy homeostasis, and have previously been reported to be seriously compromised in SOD1^G93A^ mice. Hsp70 and Hsc70, also previously described as direct interactors with mutant SOD1, were also subject to the analysis. Consequently, immunoprecipitation experiments were performed using antibodies for ATP1A, ATP5B, Hsp/Hsc70, or FLAG (Fig. 2E–I). After SDS electrophoresis, proteins were also probed for using antibodies against SOD1, ATP1A, or Hsp/Hsc70 (Fig. 2E–I). The DF mutant SOD1 but not WF protein has been shown to directly interact with ATP1A and ATP5B (Fig. 2E, F, H, and I). Our analysis also revealed a tight link between the DF mutant SOD1 and Hsp/Hsc70 (Fig. 2G).

### Immunohistochemistry

**Figure 3:**
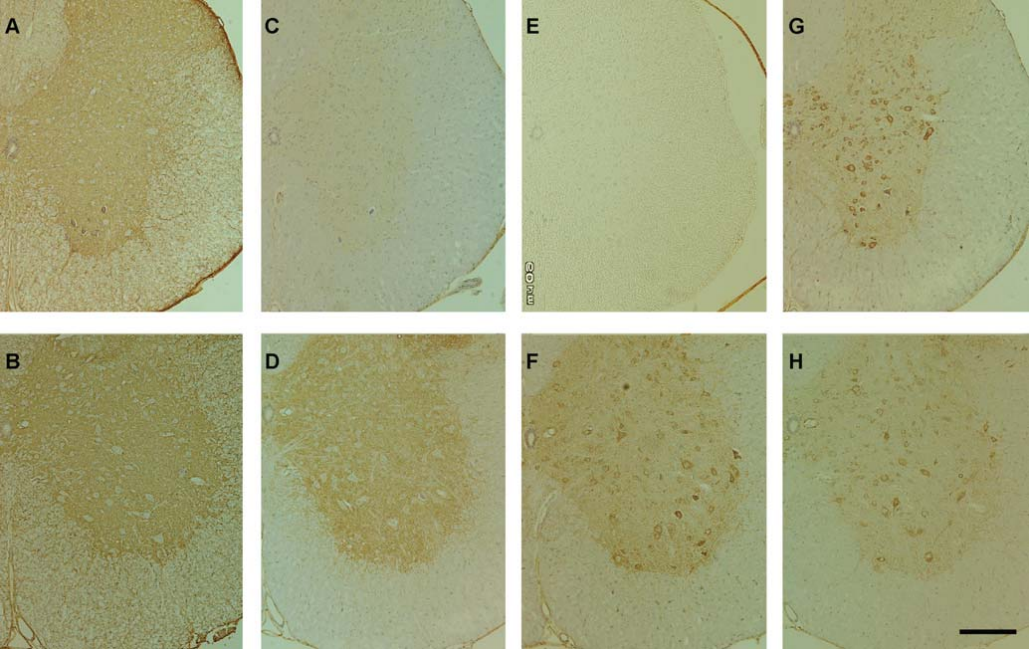
Immunohistochemistry. Fixed-frozen sections of the spinal cord (10 µm thickness) derived from symptomatic DF mice at 145 days of life (A, C, E, and G) and age-matched NTG mice (B, D, F, and H) were stained with antibodies for ATP1A (A and B), ATP1B (C and D), ATP5A (E and F), ATP5B (G and H). The tissue was counterstained with hematoxylin. Bar: 200 µm.

Immunohistochemical analysis was performed using the aforementioned antibodies for ATP1A and ATP5B, as well as for a number of respective subunits: Na/K ATPase subunit β (ATP1B) and ATP synthase α subunit (ATP5A). Immunoreactivity for ATP1A was similar in both the DF (Fig. 3A) and NTG (Fig. 3B) mice. Meanwhile, immunoreactivity for ATP1B was invariably decreased in DF mice (Fig. 3C) compared to the NTG mice (Fig. 3D).

As ATP5A and ATP5B are the major components of the mitochondrial ATP synthase, spotty expression is seen in the cell body in NTG mice (Fig. 3E and G). Reduced expression of ATP5A was evident in the DF mice (Fig. 3F), while the expression of ATP5B was unaltered between both groups (Fig. 3G and H).

## Discussion

We first hypothesized that there could be a specific ligand protein(s) for the mutant SOD1 only in the spinal cord and motor cortex. However, the results instead revealed that the mutant SOD1-interacting proteins were rather ubiquitously expressed. As Rakhit et al. suggested [12], the hydrophobic residues of the mutant SOD1, normally buried in the dimer interface, would be responsible for the interaction with a broad spectrum of proteins. Also unexpectedly, many of the mutant SOD1-interacting proteins were involved in ATP production and utilization (Table 1). Because ATP is the “energy currency of the cell” through which a diverse range of cellular activities are mediated, decreased ATP production and/or inability of ATP utilization inevitably cause devastating consequences for the cell. Among those ATP-related molecules, we especially focused on two ATPases: Na/K ATPase and ATP synthase. It is not only because they show high probability scores and high peptide hits in the proteomic analyses, but also because Na/K ATPase and ATP synthase are the largest ATP consumers and largest ATP producers in the cell, respectively. Moreover, as described later, the impairment of the ATPases is methodologically endorsed by a number of previous observations using the SOD1^G93A^ mouse model, a standard murine ALS model. So far there is no sufficient explanation elucidating the connection between mutant SOD1 and these ATPases. The direct protein-protein interactions presented here should be a convincing interpretation.

### Na/K ATPase

Previous reports indicate that Na/K ATPase enzymatic activity decreases significantly in the motor neurons of SOD1^G93A^ mice and chronically from the presymptomatic stage until the end stage [16]. Another report suggests that reduction in Na/K ATPase activity extends throughout the spinal cord of SOD1^G93A^ mice [17]. Na/K ATPase consumes 50% of the energy supply in the CNS [18], [19]. Harnessing the energy of ATP hydrolysis, the Na/K antiporter transports K+ ions into and Na+ ions out of the cell. This creates an electrochemical gradient across the plasma membrane that secondarily enables the transportation of ions, amino acids, glucose, and so on. Inhibition of the Na/K ATPase causes neurons to become incapable of maintaining their cellular volume, resulting in neuronal swelling [20]. It is reported that damage to the Na/K ATPase itself and/or subsequent impairment by the subnormal ATP production render neurons more sensitive to glutamate excitotoxicity [2], [17], [21], [22]. The Na/K ATPase consists of an α (ATP1A) and a β (ATP1B) subunit. The ATP1A, which is the catalytic subunit where ATP binding sites are located, is sensitive to damage by free radicals and other oxidative stresses [17], and it is also a target of peroxynitrite [20]. In the CNS, α1 is ubiquitous, while α2 is expressed in glial cells and α3 is expressed in neurons. All of these were shown to interact with DF mutant SOD1 in our experiment. Moreover, the altered immunoreactivity for ATP1B in the DF mice was clearly evident using immunohistochemical methods.

### ATP synthase

It is evident that a fraction of SOD1 is localized in the mitochondria [23], [24]. ATP synthase, the final enzyme in the respiratory chain, resides in the mitochondrial inner membrane and generates most of the ATP in aerobic tissues. Our proteomic analysis also shows that α (ATP5A), β (ATP5B) and γ subunits of ATP synthase interact with the mutant SOD1. These 3 subunits, belonging to the F1 catalytic core of ATP synthase, play a key role in energy production. Our results also indicate a tendency toward ATP reduction in the DF spinal cord. Immunohistochemical analysis revealed a marked reduction of ATP5A expression in the spinal cord of DF mice. While little is known about the role of ATP5A in FALS pathogenesis, growing evidence indicates that ATP5B is one of the stress-prone proteins in G93A mice. The quantity of ATP5B is already decreased in the spinal cord of SOD1^G93A^ mice during the presymptomatic stage [25], as well as in the mitochondrial fraction of end-stage SOD1^G93A^ mice [26]. The fact that ATP5B is overnitrated in SOD1^G93A^ mice before symptom onset may strongly influence interactions between subunits and impair the complex machinery of ATP production [27]. These results may help to explain the reduction of ATP synthesis in spinal cord mitochondria of symptomatic SOD1^G93A^ mice [23], and the marked reduction of ATP content in the cortex and spinal cord of presymptomatic SOD1^G93A^ mice [21].

### Conclusion

A wide range of proteins were shown to interact with the monomer-misfolded mutant SOD1, which is not necessarily an exceptional form in FALS-associated homodimeric mutant SOD1s. The mutant SOD1-interacting proteins might be compromised in their innate functions in a normal cellular compartment by bonding with the mutant SOD1. Among them, the impairment of Na/K ATPase and/or ATP synthase alone could cause devastating cellular consequences.

## Materials and Methods

### Transgenic mice

Two lines of transgenic mice were used in this experiment. The DF (deletion, FLAG) mice ubiquitously expressed the SOD1^L126delTT^ with a FLAG sequence at *C* terminal, as did the WF (wild type, FLAG) mice exhibiting a wild-type human SOD1 with the FLAG sequence [10] (Fig. 1). The DF mice showed ALS-like symptoms, while the WF mice did not. As a further negative control, wild-type C57BL/6 (NTG) mice were subjected to the same analysis. The homozygotes of DF mice begin to show clinical symptoms at around 140 to 147 days of age. After the disease duration of 1–2 weeks, the mice die at 154–161 days [10]. We analyzed the DF mice around the age of 145 days, beginning to show distinct motor neuron symptoms but without emaciation, as well as presymptomatic DF mice aged 35 days.

All animal experiments were carried out in accordance with Guidelines for Animal Experimentation at the Faculty of Medicine, Tottori University, and all efforts were made to minimize animal numbers and suffering during the experiments. Mice were sacrificed by intraperitoneal injection of pentobarbital. The spinal cords were rapidly removed (n = 5 in case of DF mice age 35, otherwise n = 4) and immediately stored in liquid nitrogen until use.

### Pull-down LC-MS/MS analysis

Spinal cords as well as cerebellum were suspended 1∶10 (w/v) in a lysis buffer (0.15 M NaCl, 10 mM Tris-HCl and 1% Triton X-100, pH 7.4) containing a cocktail of protease inhibitors (Roche Diagnostics, Switzerland) and homogenized [28]. After the brief (1 second, 3 times) sonication, the cell lysates were centrifuged for 15 min at 14,000 rpm to remove cell debris. Each sample contained 0.8 ml of tissue extract with a total protein concentration of between 5 and 6 mg/ml. After the pre-clear using a protein A/G plus-agarose (sc-2003, Santa Cruz Biotechnology, USA), the supernatant was incubated with an anti-FLAG M2 affinity gel (Sigma, USA). The immunoprecipitated samples were eluted with 3×FLAG peptide (Sigma). The eluted samples were digested by trypsin in a single tube and the resulting mixtures of peptides were directly subjected to the LC-MS/MS analysis system (Zaplous, AMR, Japan) using Finnigan LTQ (ThermoFisher Scientific, USA). The protein annotation data was queried in the NCBI_nr database reconstructed for mouse (Jan. 10, 2007) using Bioworks (Ver. 3.3).

### Immunoprecipitation and western analysis

CNS tissue extracts were prepared in the same way for proteomic analysis. Immunoprecipitation was performed using the anti-FLAG M2 affinity gel or otherwise with a combination (10 µg antibody/10 µl resin) of protein A/G plus-agarose and an anti-Na/K-ATPase α, rabbit polyclonal antibody (H-300, Santa Cruz biotechnology, USA) that recognizes all Na/K-ATPase α isoforms; anti-Hsp72/73 mouse monoclonal antibody (W27, Calbiochem, USA) that recognizes both Hsp70 and Hsc70; or anti-ATP synthase beta mouse monoclonal antibody (ab5432, abcam, USA). Immobilized antibody/bound antigen was collected by centrifugation (5,000 *g* for 1 min). The resin pellet was washed three times with the lysis buffer, and the antibody/antigen protein complex was eluted with 3×FLAG peptide or with 40 µl 0.1 M glycine-HCl, pH 3.0. In the pH elution, the eluted sample was neutralized by the addition of 10 µl of 1 M Tris-HCl and 1.5 M NaCl, pH 7.4.

Immunoblot was performed using a rabbit anti-Cu/Zn superoxide dismutase polyclonal antibody (SOD-101, Stressgen, USA) for both human and mouse SOD1, rabbit anti superoxide dusmutase 1 (SOD1) human specific polyclonal antibody (AB5480, Chemicon, USA), monoclonal anti-FLAG M2 antibody (Sigma), or otherwise using the aforementioned antibodies for ATP1A and Hsp/Hsc70. Aliquots of 10 µg were electrophoresed and transferred onto PVDF membranes (Hybond-P; Amersham Biosciences, UK). For chemiluminescense detection, we used a conventional ECL system (Amersham Biosciences), as well as ECL advance and rabbit IgG TrueBlot (eBioscince, USA).

### Immunohistochemistry

Symptomatic DF mice at ∼145 days of life (within a week of symptom onset) as well as age-matched NTG mice were sacrificed and perfused with 4% paraformaldehyde. Fixed-frozen sections of the spinal cord (10 µm thickness) were stained immunohistochemically by the streptavidin-biotin method using the aforementioned antibodies for ATP1A and ATP5B, as well as anti-Na/K ATPase β1 monoclonal antibody (464.9, Santa Cruz biotechnology, USA) for ATP1B, and ATP5A monoclonal antibody (15H4, Santa Cruz biotechnology, USA). The tissue was counterstained with hematoxylin.

## References

[1] Cleveland DW, Rothstein JD (2001). From Charcot to Lou Gehrig: deciphering selective motor neuron death in ALS.. Nat Rev Neurosci.

[2] Pasinelli P, Brown RH (2006). Molecular biology of amyotrophic lateral sclerosis: insights from genetics.. Nat Rev Neurosci.

[3] Rosen DR, Siddique T, Patterson D, Figlewicz DA, Sapp P (1993). Mutations in Cu/Zn superoxide dismutase gene are associated with familial amyotrophic lateral sclerosis.. Nature.

[4] Boillee S, Yamanaka K, Lobsiger CS, Copeland NG, Jenkins NA (2006). Onset and progression in inherited ALS determined by motor neurons and microglia.. Science.

[5] Takahashi K, Nakamura H, Okada E (1972). Hereditary amyotrophic lateral sclerosis. Histochemical and electron microscopic study of hyaline inclusions in motor neurons.. Arch Neurol.

[6] Nakashima K, Watanabe Y, Kuno N, Nanba E, Takahashi K (1995). Abnormality of Cu/Zn superoxide dismutase (SOD1) activity in Japanese familial amyotrophic lateral sclerosis with two base pair deletion in the SOD1 gene.. Neurology.

[7] Watanabe Y, Kato S, Adachi Y, Nakashima K (2000). Frameshift, nonsense and non amino acid altering mutations in SOD1 in familial ALS: report of a Japanese pedigree and literature review.. Amyotroph Lateral Scler Other Motor Neuron Disord.

[8] Watanabe Y, Kono Y, Nanba E, Ohama E, Nakashima K (1997). Instability of expressed Cu/Zn superoxide dismutase with 2 bp deletion found in familial amyotrophic lateral sclerosis.. FEBS Lett.

[9] Watanabe Y, Kuno N, Kono Y, Nanba E, Ohama E (1997). Absence of the mutant SOD1 in familial amyotrophic lateral sclerosis (FALS) with two base pair deletion in the SOD1 gene.. Acta Neurol Scand.

[10] Watanabe Y, Yasui K, Nakano T, Doi K, Fukada Y (2005). Mouse motor neuron disease caused by truncated SOD1 with or without C-terminal modification.. Brain Res Mol Brain Res.

[11] Rakhit R, Crow JP, Lepock JR, Kondejewski LH, Cashman NR (2004). Monomeric Cu,Zn-superoxide dismutase is a common misfolding intermediate in the oxidation models of sporadic and familial amyotrophic lateral sclerosis.. J Biol Chem.

[12] Rakhit R, Robertson J, Vande Velde C, Horne P, Ruth DM (2007). An immunological epitope selective for pathological monomer-misfolded SOD1 in ALS.. Nat Med.

[13] Ross CA, Poirier MA (2004). Protein aggregation and neurodegenerative disease.. Nat Med.

[14] Palmieri F (2004). The mitochondrial transporter family (SLC25): physiological and pathological implications.. Pflugers Arch.

[15] Daugaard M, Rohde M, Jaattela M (2007). The heat shock protein 70 family: Highly homologous proteins with overlapping and distinct functions.. FEBS Lett.

[16] Martin LJ, Liu Z, Chen K, Price AC, Pan Y (2007). Motor neuron degeneration in amyotrophic lateral sclerosis mutant superoxide dismutase-1 transgenic mice: mechanisms of mitochondriopathy and cell death.. J Comp Neurol.

[17] Ellis DZ, Rabe J, Sweadner KJ (2003). Global loss of Na,K-ATPase and its nitric oxide-mediated regulation in a transgenic mouse model of amyotrophic lateral sclerosis.. J Neurosci.

[18] Ames Ar (2000). CNS energy metabolism as related to function.. Brain Res Brain Res Rev.

[19] Aperia A (2007). New roles for an old enzyme: Na,K-ATPase emerges as an interesting drug target.. J Intern Med.

[20] Golden WC, Brambrink AM, Traystman RJ, Shaffner DH, Martin LJ (2003). Nitration of the striatal Na,K-ATPase alpha3 isoform occurs in normal brain development but is not increased during hypoxia-ischemia in newborn piglets.. Neurochem Res.

[21] Browne SE, Yang L, DiMauro JP, Fuller SW, Licata SC (2006). Bioenergetic abnormalities in discrete cerebral motor pathways presage spinal cord pathology in the G93A SOD1 mouse model of ALS.. Neurobiol Dis.

[22] Albin RL, Greenamyre JT (1992). Alternative excitotoxic hypotheses.. Neurology.

[23] Mattiazzi M, D'Aurelio M, Gajewski CD, Martushova K, Kiaei M (2002). Mutated human SOD1 causes dysfunction of oxidative phosphorylation in mitochondria of transgenic mice.. J Biol Chem.

[24] Okado-Matsumoto A, Fridovich I (2002). Amyotrophic lateral sclerosis: a proposed mechanism.. Proc Natl Acad Sci U S A.

[25] Massignan T, Casoni F, Basso M, Stefanazzi P, Biasini E (2007). Proteomic analysis of spinal cord of presymptomatic amyotrophic lateral sclerosis G93A SOD1 mouse.. Biochem Biophys Res Commun.

[26] Lukas TJ, Luo WW, Mao H, Cole N, Siddique T (2006). Informatics-assisted protein profiling in a transgenic mouse model of amyotrophic lateral sclerosis.. Mol Cell Proteomics.

[27] Casoni F, Basso M, Massignan T, Gianazza E, Cheroni C (2005). Protein nitration in a mouse model of familial amyotrophic lateral sclerosis: possible multifunctional role in the pathogenesis.. J Biol Chem.

[28] Cottrell BA, Galvan V, Banwait S, Gorostiza O, Lombardo CR (2005). A pilot proteomic study of amyloid precursor interactors in Alzheimer's disease.. Ann Neurol.

